# Lateral hypothalamic melanin concentrating hormone‐expressing neurons both promote and are required for cue‐potentiated feeding

**DOI:** 10.1111/jne.70210

**Published:** 2026-06-04

**Authors:** Lauren M. Raycraft, Bingxin Mo, Allison Doneth, Jenna Lee‐Tanner, Alexander W. Johnson

**Affiliations:** ^1^ Department of Psychology Michigan State University East Lansing Michigan USA; ^2^ Neuroscience Program Michigan State University East Lansing Michigan USA

## Abstract

Food‐related stimuli can promote feeding behaviors independent of metabolic need. Cue‐potentiated feeding (CPF) studies in rodents offer the potential to reveal the psychobiological mechanisms underlying learned overeating behaviors. We examined whether lateral hypothalamic area (LHA) cells expressing the feeding signal Melanin Concentrating Hormone (MCH) are sufficient and necessary for CPF. Tg(Pmch‐Cre) mice received bilateral infusion of the Cre‐dependent inhibitory DREADD (hM4Di), or optrode placement together with the excitatory opsin ChR2. Mice received food deprivation by restricting access to two daily meal pellets. Once weight reached ~90% from baseline, Pavlovian training commenced, in which a tone or noise conditioned stimulus (CS+) predicted the delivery of a sucrose solution, whereas a second CS− was unpaired with sucrose delivery. Following a minimum of 2 days of ad‐libitum pellet access, mice underwent CPF testing, where the amount of licking for sucrose in the presence of the CS+ and CS− cues was assessed. In both the chemogenetic and optogenetic studies, control mice displayed CPF as evidenced by increased lick rate during the CS+ compared to the CS−. hM4Di‐mediated inhibition of LHA MCH cells significantly impaired CPF through disrupting the capacity for the CS+ to increase the mean size of licking bursts, a measure thought to index the orosensory taste properties of sucrose. By contrast, optogenetic stimulation of LHA MCH cells enhanced CPF relative to eYFP‐treated controls. Our findings support a critical role for MCH‐expressing neurons in the LHA in learned overeating behavior.

## 1 INTRODUCTION

Stimuli that are predictive of food availability can promote food intake independent of energetic need, enhancing susceptibility to weight gain in the obesogenic environment.^1^ This capacity for food cues to facilitate food intake even when not hungry can be examined using cue‐potentiated feeding (CPF), in which a food‐paired conditioned stimulus (CS) acquires motivational properties such that it can potentiate consumption of food in a sated condition.^2^, ^3^, ^4^ Accordingly, CPF offers the potential to uncover the behavioral and neurobiological mechanisms of learned overeating behaviors.

The lateral hypothalamic area (LHA) has traditionally been viewed as the brain's feeding center^5^ following the seminal discovery in the mid‐20th century that electrical stimulation of this region resulted in eating behavior^6^, whereas lesions to it led to chronic hypophagia.^7^ More recent characterizations emphasize the LHA as a molecularly diverse system that houses a variety of neuropeptide systems responsible for energy regulation.^5^, ^8^ Of these, Melanin Concentrating Hormone (MCH) has received attention as a critical feeding signal.^9^, ^10^, ^11^ MCH is synthesized in the LHA and binds to G protein‐coupled MCH receptors, MCH‐1R and MCH‐2R.^12^, ^13^ In several species (e.g., primates, dogs and ferrets) the action of MCH‐2R is preserved, however in rodents it is either non‐functional or lacking.^14^, ^15^ LHA MCH‐expressing cells project widely throughout the rodent brain^16^ and have their biological actions through MCH‐1R,^13^, ^17^ including in brain regions known to play a role in cue‐potentiated feeding (e.g., amygdala^18^) and reward‐related feeding behaviors (e.g., nucleus accumbens^19^).

Previous studies from our laboratory indicated that gene‐targeted deletion of MCH‐1R disrupted the capacity for a Pavlovian CS+ to gain conditioned reinforcing value when paired with food,^20^ as well its capacity to evoke CPF.^21^ In the current series of studies, we used chemogenetic and optogenetic approaches to respectively inhibit and stimulate LHA MCH‐expressing cells in mice during CPF testing. We first used hM4Di‐inhibitory DREADDs to inactivate LHA MCH cells during CPF. In a seperate group of mice, we implemented acute optogenetic approaches to selectively stimulate via ChR2, LHA MCH‐expressing cells during discrete episodes of CPF testing. We also examined a variety of other Pavlovian conditioned responses and analyses of licking microstructure to determine the nature of LHA MCH cell manipulations on appetitive feeding behaviors. Our findings confirm that LHA MCH neurons play a pivotal role in modulating feeding behavior independent of energetic need.

## 2 EXPERIMENT 1: CHEMOGENETIC INHIBITION OF LHA‐MCH CELLSTg(Pmch‐Cre)

### 2.1 Methods

#### 2.1.1 Subjects

Tg(Pmch‐Cre) male mice in which Cre is driven by a ~108­kb fragment of the pMCH gene promoter received stereotaxic surgery at approximately 10–12 weeks of age. Following recovery from surgery, mice were pair housed and maintained on a standard lab chow diet containing 17% fat, 54% carbohydrate and 29% protein (Teklad 2918, Indianapolis, IN) under a 12:12 dark–light cycle with lights off at 7:00 PM. All behavioral testing took place between the hours of 9:00 AM and 1:00 PM Two weeks following surgery, mice received food deprivation, which was accomplished by restricting access to two daily meal pellets. Once the weight of all mice reached ~90% from baseline, behavioral training commenced. Training and testing were conducted under the auspices of the Michigan State University Institutional Animal Care and Use Committee.

### 2.2 Viral infusions

Mice (*n* = 16) were anesthetized with 4% isoflurane exposure in oxygen and placed into a stereotaxic apparatus adapted for mouse surgeries (Stoelting, Wood Dale, IL). Subjects received bilateral injections (0.5 μL/side at a rate of 0.25 μL/min) of the inhibitory Cre‐dependent DREADD AAV8‐ pAAV‐hSyn‐DIO‐hM4D(Gi)‐mCherry (hM4Di) (Addgene, Watertown, MA; plasmid #44362) into the LHA (−1.5 mm posterior from Bregma, −5.2 mm from the dorsal surface, ±1.1 mm lateral). On completion, mice were first single housed to recover from surgery, and 3–4 days after pair housed with a previous cage littermate. To confirm viral targeting, mice were euthanized and tissue stained using published methods.^22^, ^23^, ^24^ Subsequently, fluorescence expression was traced for each mouse and layers were collapsed across subjects within each coronal plane (Bregma: −1.07, −1.23, −1.31, −1.43, −1.55, −1.67, −1.91, −2.15)^25^ producing ‘heat maps’, in which lighter and darker shading respectively indicated minimal and maximal expression.

### 2.3 Apparatus

Pavlovian training and cue‐potentiated feeding took place in eight individual Med‐associate chambers (Med Associates, St Albans, VT, USA) that were 53 × 35 × 35 cm (LWH) and contained aluminum front and back walls, clear polycarbonate sides, and a floor made of 17.8‐mm stainless steel rods spaced 0.5 cm apart. Auditory stimulus presentation was triggered by the activation of an audio generator programmed to emit a 3 kHz tone or white noise (each 80 dB). In addition, a clicker was mounted outside of the chamber on the wall adjacent to the food magazine and was used to initially signal reward availability. Chamber illumination was provided by a 28 V, 100 mA red house light mounted on the inside wall of the sound‐attenuating chamber. The boxes were modified such that the food cup of each chamber contained a custom‐built liquid well into which solutions could be delivered via an external syringe pump that was connected through tubing.^26^ Housed within the liquid well was a custom‐built lickometer, through which fiber optics were used to introduce a light beam via the fluid‐air interface of a fluid bolus. This permitted measuring individual licks through disturbances in the light surface at the interface when the fluid was contacted. Fluid in the liquid well could be suctioned off at the end of each rewarded trial through a vacuum that was connected by tubing at the bottom of the food well and activated by the release of an attached solenoid. The food cup magazine also contained an infrared photocell to enable monitoring of the time spent within the magazine and the number of head entries made into it. An IBM‐compatible computer equipped with Med‐PC software controlled and recorded all stimuli and responses.

### 2.4 Food‐cup training

Mice were placed into their assigned chamber with a 10% sucrose (w/v) reward immediately available. The mouse entering the food cup triggered the initiation of 16 deliveries of 50 μL of 10% sucrose, with each delivery occurring via a random time interval of 120 s and coinciding with a magazine clicker that was activated for 0.25 s. Sessions took approximately 30–45 min to complete, and mice had two single sessions conducted over a 2‐day period. At the conclusion of the second day of food cup training, all mice reached the criterion of at least 10 s of time in the magazine with the reward present.

### 2.5 Pavlovian training

**FIGURE 1:**
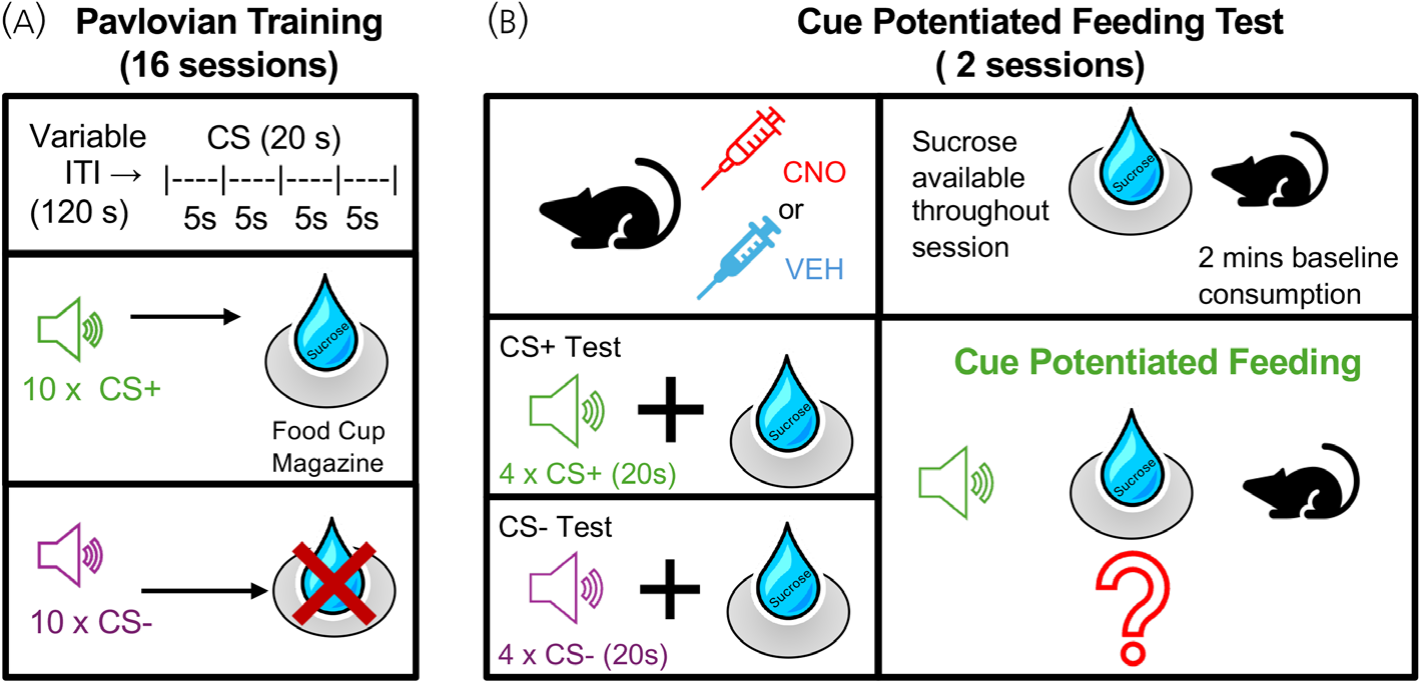
Simplified schematic of the training and testing parameters for cue potentiated feeding. (A) Food deprived mice received 16 Pavlovian training sessions in which sucrose was delivered during each 20 s CS+ but not CS− trial, which were separated by the intertrial interval (ITI). Mice received a total of 10 CS trials per session. (B) Following at least 2 days ad‐libitum access to laboratory chow, mice received an injection of either clozapine‐N‐oxide (CNO) or vehicle (VEH) prior to a 2 min baseline sucrose consumption test and subsequent cue potentiated feeding testing. Mice received separate tests with the CS+ and CS− cues, wherein sucrose was available throughout testing. Cue potentiated feeding reflects an increase in sucrose licking during the CS+ but not CS− trials.

Mice received a single Pavlovian conditioning session each day for a total of 16 sessions (Figure 1A). During each session, mice received presentations of 10 reinforced conditioned stimulus + (CS+) trials, and 10 non‐reinforced conditioned stimulus—(CS−) trials. Each trial consisted of a 20 s presentation of either a tone or white noise stimulus separated by a variable intertrial interval (ITI) of 120 s. Sucrose was delivered only during CS+ trials and occurred in a pseudorandom manner. This was achieved by separating each CS+ into four 5 s epochs. For the first 5 s epoch, sucrose delivery occurred on a maximum of three and a minimum of two (out of the 10) trials per session. The remaining epochs were programmed such that sucrose delivery occurred in two of the four epochs per trial. The delivery of sucrose across trials was randomized each day. Subsequently, CS+ time (i.e., percent time in the food cup) was calculated from the first 5 s epoch during the seven trials where sucrose was not delivered and this was used as a measure of conditioning that was uncontaminated by sucrose delivery. Following three sessions of Pavlovian conditioning, the magazine clicker was removed. On completion of 16 Pavlovian conditioning sessions, mice were provided 2 days (3 nights) of *ab‐libitum* access to their standard laboratory diet with the goal of restoring subjects closer to their original baseline weight.

### 2.6 Cue‐potentiated feeding

Fifteen minutes prior to the start of the session, mice were injected with their assigned dose of either vehicle or clozapine‐N‐oxide (CNO). Mice then received CPF testing with either the CS+ or CS− presented separately on subsequent days of testing (test order counterbalanced) (Figure 1B). For each test session the sucrose reward was available for consumption at all times. At the start of the session, 50 μL of sucrose was available in the food cup, and additional 50 μL deliveries occurred every 40 licks as mice consumed the liquid. Test sessions began with a 2‐min baseline consumption period. This was followed by one of four test trials during which either the tone or the noise stimulus was presented for 20 s. Each trial was preceded by a fixed 2‐min ITI. Mice were then returned to their homecage and received *ab‐libitum* access to their standard laboratory diet. The next day, mice received the alternative CS with drug administration and testing identical to the first test session.

### 2.7 Drugs

CNO stock solution was made by dissolving in 10% (2‐Hydroxypropyl)‐β‐cyclodextrin and 0.2 M Phosphate‐buffered saline (PBS). CNO working solution was prepared fresh daily and delivered by intraperitoneal injection at a dose of 0.3 mg/kg. Mice received injections of either CNO (*n* = 8) or vehicle (0.2 M PBS) (*n* = 8) at a volume of 10 mL/kg.

### 2.8 Licking microstructure analysis

For licking microstructure analyses, the size and number of licking bursts were examined.^27^, ^28^ A licking burst was defined as two or more consecutive licks, with pauses greater than 1 s determining the licking burst termination. The burst number reflected the initiation of licking behavior following a 1 s pause. The mean burst size is thought to reflect the orosensory and hedonic evaluation of a stimulus, whereas the number of bursts initiated reflects post‐ingestive negative feedback and motivationally oriented behaviors.^28^, ^29^

### 2.9 Data analysis

For Pavlovian training, the primary outcome measure was the percent time spent in the food cup during the non‐reinforced first epoch of the CS+ and CS− trials, which was averaged for each session. The 16 sessions were further averaged into 4 session blocks and a three‐way mixed repeated measures ANOVA with variables of group (vehicle, CNO) × cue (CS+, CS−) × session block (1–4) was utilized. A similar analysis was conducted on the pre‐CS data, which reflected the percentage of time in the food cup in the 20 s prior to the CS+ and CS− trials. Follow‐up cue × session block ANOVAs were also conducted.

For cue‐potentiated feeding the 2 min baseline licks/min were analyzed using a two‐way mixed ANOVA with factors of group and test. The licks/min data for the CS+ and CS− tests were the data of primary interest and were examined with a three‐way mixed repeated measures ANOVA with variables of group (vehicle, CNO) × test cue (CS+, CS−) × trial (1–4). The ITI data for the CS+ and CS− tests were analyzed in a similar manner. For licking microstructure analyses, a two‐way group × test cue ANOVA was implemented for the mean burst size and burst number analyses. The percentage of time spent in the food cup and food cup entries/min for the CS+ and CS− trials during the cue and the preceding ITI periods were analyzed in an identical manner to the licks/min data. Follow‐up group × trial and test cue × trial ANOVAs were also implemented. The α level for significance was .05 and all analyses were conducted using Statistica (Statsoft, Tulsa, OK).

## 3 RESULTS

### 3.1 Immunofluorescence

**FIGURE 2:**
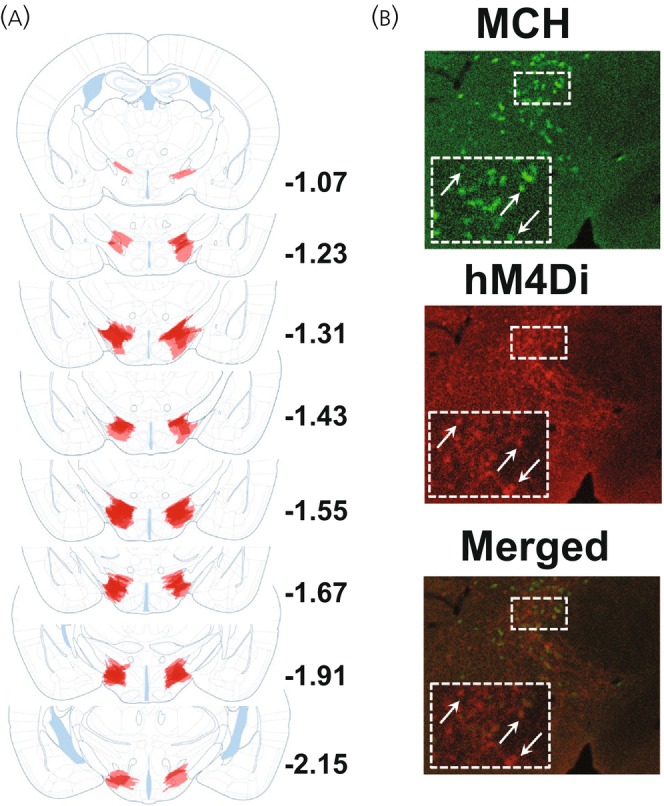
Expression of DREADDs in LHA. (A) hM4Di expression in LHA of Tg(Pmch‐Cre) mice. Lighter and darker shading respectively indicate minimal and maximal expression at each coronal plane. (B) Overlap between MCH protein (green) and hM4Di (red) in LHA. Arrows indicate somatic infection.

hM4Di‐expression extended throughout the rostral‐caudal axis of the LHA and was most densely observed in the dorsolateral LHA (Figure 2). A subset of vehicle and CNO mice was removed from the study due to either insufficient hM4Di expression (*n* = 3) or unilaterally restricted transfection (*n* = 2). This resulted in *n* = 6 vehicle treated mice and *n* = 5 CNO treated mice that were subsequently used for the analyses.

### 3.2 Pavlovian training

**FIGURE 3:**
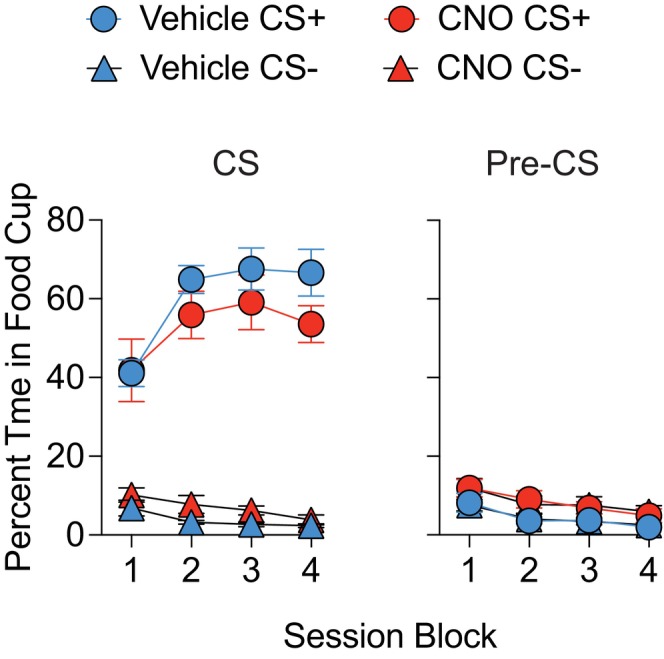
Percentage time in food cup during Pavlovian acquisition. Tg(Pmch‐Cre) hM4Di‐treated mice that subsequently received vehicle or CNO prior to cue‐potentiated feeding testing displayed similar rates of acquisition to the CS+ relative to the CS− (left panel). Pre‐CS responding prior to each CS was comparable between the two groups. Each session block reflects the mean of 4 training sessions. Error bars indicate standard error of the mean (SEM).

Pavlovian acquisition revealed that mice subsequently assigned to vehicle or CNO at CPF testing showed similar increases in the percent time spent in the food cup during CS+ presentation as training progressed (Figure 3), whereas time spent during CS− was consistently low throughout training. ANOVA confirmed this impression as it revealed a main effect of cue (F (1, 9) = 238.52, *p* < .0001), session block (F (3, 27) = 10.16, *p* = .0001), and an interaction between the two variables (F (3, 27) = 23.97, *p* < .0001). Separate tests of simple main effects on the two‐way cue × session block ANOVA revealed that mice spent significantly more time in the food cup during CS+ compared to CS− across all session blocks (smallest F‐value; block 1, F (1, 10) = 58.58, *p* < .0001). The analysis of pre‐CS responses prior to each cue revealed a main effect of session block only (F (3, 27) = 23.79, *p* < .0001). These findings confirm that prior to chemogenetic inhibition, all groups of mice acquired the simple Pavlovian discrimination in a comparable manner.

### 3.3 CPF test: Food cup responses

**FIGURE 4:**
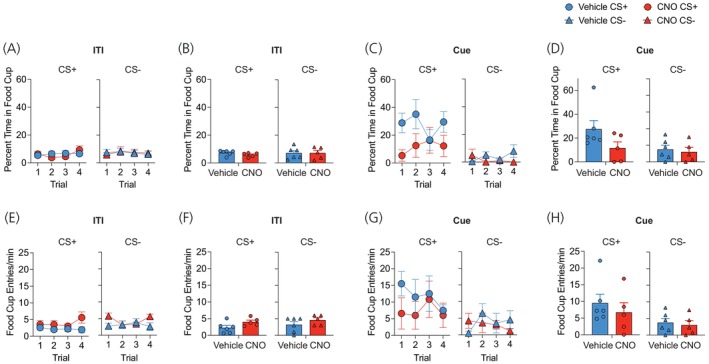
Food cup responses during cue‐potentiated feeding testing. (A) ITI food cup responses during the CS+ and CS− tests separated across trials and (B) averaged across the test session. (C) Percentage of time in food cup across trials and (D) averaged across the test session for CS+ and CS− tests. (E) Entries/min to food cup during ITI for CS+ and CS− trials and (F) across session. (G) Entries/min to the food cup across CS trials and (H) test session means.

As a result of the return to ad‐libitum feeding, the weight of vehicle‐ (t (5)=1.28, *p* = .26) and CNO‐treated (t (4) =2.20, *p* = .09) hM4Di Tg(Pmch‐Cre) mice at test did not significantly differ from baseline (Table 1). During cue‐potentiated feeding, the percentage of time spent in the food cup during the ITI period was generally low (Figure 4A,B), with ANOVA revealing no main effects or interactions between drug or test (F's < 1.6; *p*'s > .21). Conversely, with CS presentations, mice spent significantly more time during the CS+ compared to the CS− (Figure 4C,D). ANOVA revealed a main effect of test cue only (F (1, 9) = 15.34, *p* = .01). For the rate of entry to the food cup, this was also low during the ITI period (Figure 4E,F) and no significant main effects or interactions observed (Largest F‐value; drug, (F (1, 9) = 3.18, *p* = .1)). Alternatively, CS+ presentation elicited higher rates of food cup entry compared to the CS− (Figure 4G,H), and ANOVA revealed a main effect of test cue only (F (1, 9) = 8.89, *p* = .01).

**TABLE 1:** Body weights prior to food deprivation (baseline), the final training session, and CPF testing.

Group	Baseline	Final training session	CPF Test 1	Baseline vs. training	Baseline vs. CPF
CNO	28.0 ± 1.48	25.8 ± 1.46	26.8 ± 1.72	t = 11.1, df = 4, *p* < .001	t = 2.20, df = 4, *p* = .09
Vehicle	31.0 ± 1.6	28.5 ± 1.34	30.4 ± 1.54	t = 9.05, df = 5, *p* < .001	t = 1.28, df = 5, *p* = .26
eYFP	28.6 ± 1.67	26.1 ± 1.62	28.0 ± 1.72	t = 7.55, df = 3, *p* < .01	t = 1.01, df = 3, *p* = .38
ChR2	26.2 ± 2.06	24.2 ± 1.68	25.4 ± 1.9	t = 4.52, df = 5, *p* < .01	t = 2.15, df = 5, *p* = .08

### 3.4 Test: Licking responses

**FIGURE 5:**
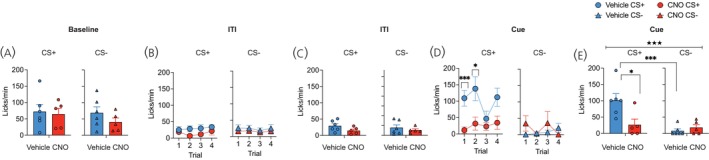
Licking responses during cue‐potentiated feeding test. (A) Lick rates during the 2 min baseline consumption phase were comparable between groups and tests. (B, C) ITI lick rates (B) prior to each trial or (C) averaged across ITI trials did not differ between groups or tests. (D) Presentation of the CS+ increased lick rates for vehicle‐treated mice, however chemogenetic inhibition of LHA MCH cells disrupted cue‐potentiated feeding in CNO‐treated hM4Di Tg(Pmch‐Cre) mice. Significant effect of CNO treatment, ***p = .006, *p = .01. (E) Mean lick rates averaged across test trials revealed significant increase in CS+ licks in vehicle‐treated hM4Di Tg(Pmch‐Cre) mice only. Significant cue × drug interaction ★★*p* = .005, main effect of cue ***p = .0002, main effect of drug *p = .02.

Consumption during the 2 min period prior to the initiation of test trials was comparable between the two drug groups and across tests (Figure 5A), (F's < 1.79; *p*'s > .21). Lick rates were also similar between the two groups during the ITI period for both CS+ and CS− tests (Figure 5B,C) (F's < 1.6; *p*'s > .23). However, presentation of the CS+ significantly increased licking in vehicle‐treated mice relative to CS− trials and when compared to CNO‐treated hM4Di Tg(Pmch‐Cre) mice (Figure 5D,E). For the trial data (Figure 5D), ANOVA revealed a main effect of test cue (F (1, 9) = 19.02, *p* = .001), a test cue × trial (F (3, 27) = 3.00, *p* < .05), and a cue × drug (F (1, 9) = 13.43, *p* < .01) interaction. Separate test cue × trial ANOVAs for each drug condition revealed for vehicle treated mice a main effect of test cue (F (1, 5) = 36.21, *p* = .001) and a cue × trial interaction (F (3, 15) = 3.35, *p* < .05), due to significantly elevated CS+ lick rates during the 1st and 2nd and 4th trials (smallest F‐value; trial 2, F (1, 5) = 15.32, *p* = .01). This cue‐potentiated feeding effect in vehicle‐treated mice was markedly absent in mice that received chemogenetic inhibition of LHA MCH cells (F's < 1.33; *p*'s > .31). Moreover, CS+ lick rates were significantly higher in vehicle relative to CNO‐treated mice during trial 1 (F (1, 9) = 11.27, *p* = .008) and trial 2 (F (1, 9) = 5.47, *p* < .05). The mean CS data (Figure 5E) similarly revealed a test cue × drug interaction (F (1, 9) = 13.43, *p* < .001), with vehicle (F (1, 9) = 35.42, *p* < .001) but not CNO‐treated mice (F < 1) displaying greater licking during CS+ compared to CS−, as well as significantly elevated lick rate during CS+ in vehicle compared to CNO‐treated mice (F (1, 9) = 7.28, *p* < .05).

**FIGURE 6:**
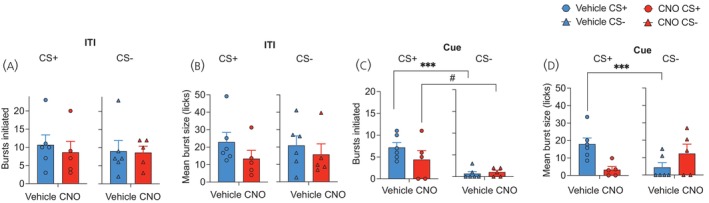
Cue‐potentiated feeding licking microstructure analyses. During the ITI period, the (A) number of bursts and (B) size of licking bursts did not differ between the drug conditions or test cue. (C) CS+ presentation increased burst number for both vehicle and CNO‐treated hM4Di‐treated Tg(Pmch‐Cre) mice. Main effect of cue, ***p = .001, #*p* = .05. Conversely, (D) increased mean burst size during CS+ was only revealed in vehicle‐treated mice. ***p = .007.

The analysis of licking microstructure revealed that both the number of bursts (Figure 6A) and the mean size of licking bursts (Figure 6B) were similar during the ITI for both the CS+ and CS− tests and did not differ as a function of CNO treatment (F's < 1.04; *p*'s > .33). During the cue trials, CS+ presentation increased the number of licking bursts in both groups compared to CS− trials (Figure 6C), as the ANOVA revealed a main effect of test cue only (F (1, 9) = 22.54, *p* < .001). On the other hand, the mean size of licking bursts was significantly elevated in vehicle‐treated mice during the CS+ compared to CS−, whereas chemogenetic inactivation of LHA MCH cells drastically diminished this effect (Figure 6D). ANOVA revealed a test cue × drug interaction (F (1, 9) = 9.43, *p* = .01) due to a significantly elevated burst size during CS+ in vehicle compared to CNO‐treated Tg(Pmch‐Cre) mice (F (1, 9) = 11.87, *p* = .007).

## 4 EXPERIMENT 2: OPTOGENETIC STIMULATION OF LHA‐MCH CELLS

### 4.1 Methods

#### 4.1.1 Subjects and apparatus

**FIGURE 7:**
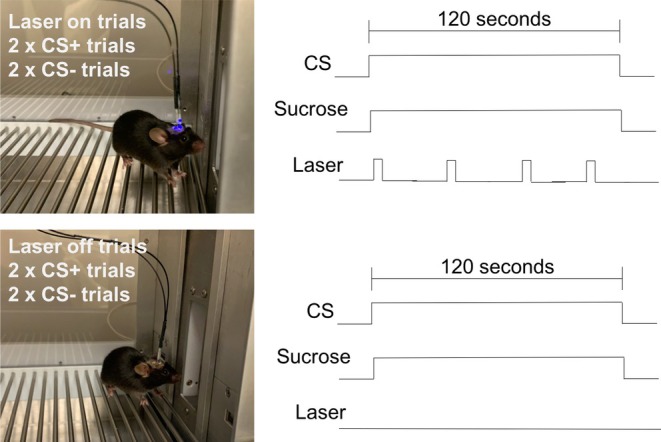
Simplified schematic of the optogenetic testing of Tg(Pmch‐Cre) mice. During cue potentiated feeding tests, each CS was presented for 120 s, and sucrose was available for the duration of testing. During laser on ‘stimulated’ trials, mice received laser stimulation (5 s on/off, 473 nm, 5 ms pulses at 20 Hz and power of ~20 mW), whereas during laser off ‘non‐stimulated’ trials, the laser was not activated. All mice received 2 stimulated and 2 non‐stimulated trials with the CS+ and CS− cues.

Twelve week old, Tg(Pmch‐Cre) male mice (*n* = 14) were used and housed in an identical manner to Experiment 1, with the exception that after surgery, mice remained individually housed for the duration of the study. Mice in this study were also tested with the same behavioral apparatus, with the addition of a swivel arm and gimble inserted into the test chamber to house the fiber optic used for optogenetic stimulation (Figure 7).

### 4.2 Viral infusion and optrode placement

Anesthesia and post‐operative care were the same as Experiment 1. Tg(Pmch‐Cre) mice received bilateral injections (0.5 μL/side at a rate of 0.25 μL/min) of either the Cre‐dependent channel rhodopsin ChR2 (*n* = 10) (pAAV‐EF1a‐double floxed‐hChR2 (H134R)‐EYFP‐WPRE‐HHGHpA) (Addgene, plasmid #20298) or control eYFP (*n* = 4) (pAAV‐Ef1a‐DIO‐EYFP) (AAV5) (Addgene, plasmid #27056) into the LHA (−1.5 mm posterior from Bregma, −5.2 mm from the dorsal surface, ±1.1 mm lateral). In addition, optical fiber ferrule tips (200 μm core, 4.1 mm; Thorlabs) were placed dorsal (−4.6 mm) to the injection site and affixed with dental acrylic (Lang Dental Manufacturing Company). On completion of the study, tissue was harvested and viral expression and ferrule tip placement quantified. At this time, *n* = 3 ChR2 mice were excluded due to an absence of ChR2 expression in at least one hemisphere.

### 4.3 Food cup training and tether habituation

Mice received two food cup training sessions with 10% sucrose, identical to the previous experiment. However, between the two training sessions, mice received three sessions aimed at habituating the mice to contemporaneous tethering and reward consumption. To achieve this, on separate days mice were tethered once unilaterally on the left and right sides (habituation sessions 1–2) and once bilaterally (habituation session 3). These sessions occurred in a distinct context, with laminated black and white checkered contextual stimuli placed on the interior walls and a vanilla scented odor emanating through the chamber. In these 10 min tests, mice were free to consume an unrestricted amount of a distinct 20% (w/v) polycose solution with the aim of a minimum of 200 licks during the session.

### 4.4 Pavlovian training and Cue‐potentiated feeding

A handful of modifications in the training protocol were implemented for Experiment 2. First, we extended the CS duration to 120 s (four 30 s epochs) to allow for a more prolonged interval to examine licking during CPF testing with optogenetic stimulation. Second, the number of training sessions was reduced to 10, in an attempt to produce a weaker form of cue‐potentiated feeding and thus limit the potential of ceiling effects in lick rate during optogenetic testing. For CPF testing, mice were tethered and received laser stimulation (ThorLabs, Newton, NJ) (5 on/off, 473 nm, 5 ms pulses at 20 Hz and power of ~20 mW) via triggering of a waveform generator (Agilent Technologies, Santa Clara, CA) using the Med Associates interface. Laser stimulation occurred only during the CS+ and CS− cue presentations (not ITI). Furthermore, two of the trials were designated as laser stimulated (e.g., Trials 1 and 3), whereas in the remaining non‐stimulated trials (e.g., Trials 2 and 4) the laser was not activated (Figure 7). The designation of trial stimulation was fully counterbalanced across viral conditions and tests. This design allowed for a rigorous within‐subject analysis of optogenetic evoked stimulation of MCH‐expressing cells during CPF. During the CPF test, one of the ChR2 mice lost its head stage and was immediately euthanized and removed from the study, resulting in a total of *n* = 6 ChR2 mice.

### 4.5 Data analysis

For Pavlovian conditioning, percentage time during the CS (the primary outcome measure) and pre‐CS periods was analyzed using a three‐way mixed ANOVA with virus (ChR2, eYFP), cue (CS+, CS−), and session (1–10) as variables. CPF test baseline consumption data were subjected to a virus × test cue ANOVA, whereas lick rates (licks/min; primary outcome measure) and other secondary measures including percent time in food cup and food cup entries/min during the CS+ and CS− tests were analyzed with a test cue × virus × stimulation (stimulated trials, non‐stimulated trials) mixed ANOVA. Follow‐up test cue × stimulation and virus × stimulation ANOVAs were utilized to explore significant interactions.

## 5 RESULTS

### 5.1 Immunofluorescence

**FIGURE 8:**
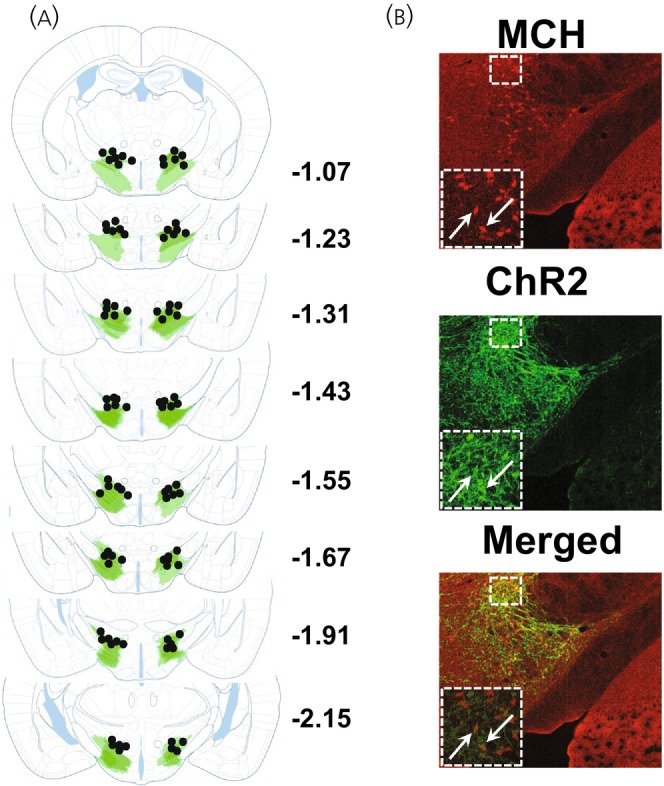
Expression of ChR2 in LHA. (A) ChR2 expression and optrode placement in LHA of Tg(Pmch‐Cre) mice. Lighter and darker shading respectively indicate minimal and maximal expression at each coronal plane. (B) Overlap between MCH protein (red) and ChR2 (green) in LHA. Arrows indicate somatic infection.

ChR2‐expression extended broadly throughout the LHA including perifornical and dorsolateral subregions (Figure 8).

### 5.2 Pavlovian training

**FIGURE 9:**
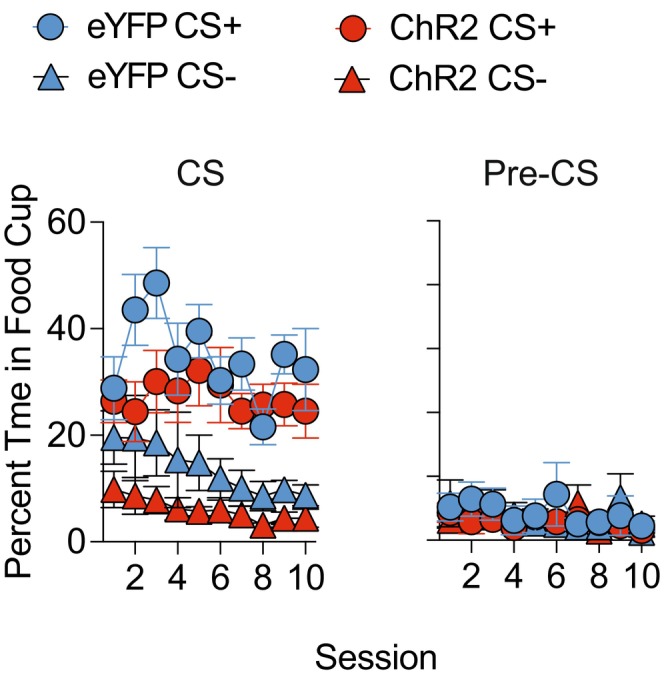
Percentage time in food cup during Pavlovian acquisition. Both eYFP and ChR2‐treated Tg(Pmch‐Cre) mice displayed similar rates of acquisition to the CS+ relative to the CS− (left panel). Pre‐CS responding prior to each CS was also comparable between the two groups.

Throughout training, mice in both viral groups spent significantly more time in the food cup during CS+ compared to CS− trials (Figure 9). ANOVA revealed a main effect of cue (F (1, 8) = 37.8, *p* < .001) and session (F(9, 72) = 3.51, *p* < .01) only. During the pre‐CS, the percentage of time spent in the food cup did not change or differ between cues or viral conditions (F's < 1.33; *p*'s > .23).

### 5.3 CPF test: Food cup responses

**FIGURE 10:**
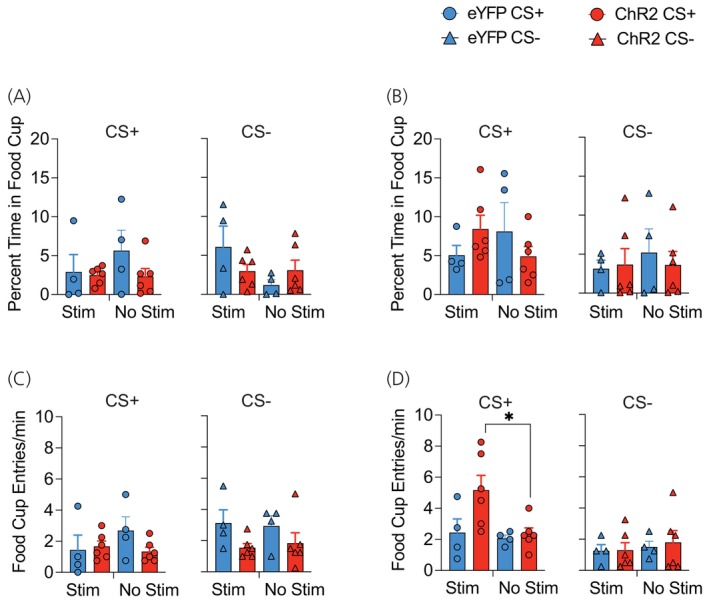
Food cup responses during cue‐potentiated feeding test and acute optogenetic stimulation. (A, B) The percentage of time in the food cup was comparable during the (A) intertrial interval and (B) cue presentation trials irrespective of viral condition or test. (C) Entries/min during the intertrial interval were similar across mice for the CS+ and CS− tests. (D) Optogenetic stimulation increased the rate of food cup entry in Tg(Pmch‐Cre) mice, main effect of stimulation, *p* < .05.

Ad‐libitum access to laboratory chow prior to CPF testing resulted in similar body weight at test compared to baseline in both eYFP (t (3) = 1.01, *p* = .38) and ChR2 (t (5) = 2.15, *p* = .08) mice (Table 1). The percentage of time spent in the food cup during the ITI did not differ as a function of virus, test cue, or upcoming stimulation (F's < 1.04; *p*'s > .33) (Figure 10A). For the percentage of time during CS presentations (Figure 10B), ANOVA revealed a main effect of test cue only (F (1, 8) = 33.53, *p* < .0001). ITI food cup entries (Figure 10C) were similar across the CS+ and CS− test for eYFP and ChR2 mice (*p*'s > .11). During the cue presentations (Figure 10D), three‐way ANOVA revealed a main effect of test cue (F (1, 8) = 22.19, *p* < .001) and a test cue × stimulation interaction (F (1, 8) = 6.42, *p* < .05). Separate cue × stimulation ANOVAs for each viral condition revealed no main effects or interactions in eYFP mice (Largest F‐value; cue, F (1, 3) = 4.61, *p* = .12); however, in ChR2‐treated mice, a test cue × stimulation interaction was revealed (F (1, 5) = 8.19, *p* < .05). This was due to greater food cup entries in ChR2 mice during the CS+ compared to CS− during stimulated (F (1, 5) = 55.65, *p* < .001) but not non‐stimulated trials (F < 1) and elevated CS+ entry rate following LHA MCH stimulation compared to non‐stimulated CS+ trials (F (1, 5) = 10.27, *p* < .05).

### 5.4 CPF test: Licking responses

**FIGURE 11:**
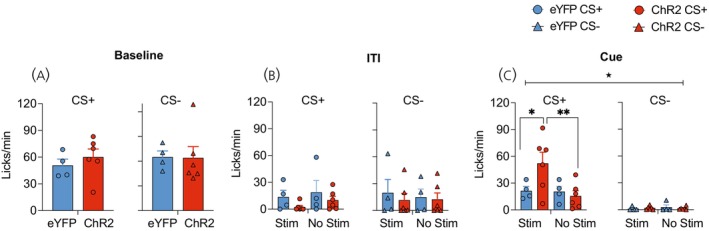
Licking responses during cue‐potentiated feeding test and acute optogenetic stimulation. (A) Lick rate (licks/min) during the baseline consumption test was comparable between the two viral conditions. (B) Licking during the intertrial interval was similar during the CS+ and CS− tests and between eYFP and ChR2 treated mice. (C) Optogenetic stimulation of LHA MCH cells promoted licking for sucrose during the CS+ but not CS− test. ★ indicates significant three‐way interaction (*p* = .01). *Significant difference between viral conditions, *p* < .05, **main effect of stimulation, *p* < .01.

Lick rates during the baseline period of consumption were comparable across viral conditions and test (Figure 11A) (F's < 1). In addition, lick rates were also comparable across subject conditions, upcoming stimulation and cue during the ITI (Figure 11B) (F's < 1.72; *p*'s > .22). However, during the CPF test, optogenetic stimulation during the CS+ enhanced licking for sucrose in ChR2‐treated Tg(Pmch‐Cre) mice (Figure 11C). ANOVA revealed a main effect of test cue (F (1, 8) = 17.5, *p* < .001), stimulation (F (1, 8) = 8.99, *p* = .01), a test cue × stimulation (F (1, 8) = 13.87, *p* = .005), a virus × stimulation (F (1, 8) = 9.81, *p* = .01), and a significant three‐way interaction between all the variables (F (1, 8) = 10.41, *p* = .01). Follow‐up test cue × stimulation ANOVAs revealed in eYFP mice a main effect of cue only (F (1, 3) = 16.39, *p* = .02), indicating that CPF occurred as expected in control mice. In ChR2 mice, the ANOVA revealed a main effect of test cue (F (1, 5) = 11.58, *p* = .01) as well as stimulation (F (1, 5) = 21.5, *p* < .01) and a significant interaction between the two variables (F (1, 5) = 26.01, *p* = .003). This in part reflected an increase in lick rate during the CS+ between stimulated and non‐stimulated trials (F (1, 5) = 23.9, *p* < .01), and a significant difference in CS+ compared to CS− lick rate during stimulated (F (1, 5) = 15.37, *p* = .01) but not non‐stimulated trials (F (1, 5) = 5.18, *p* = .07).

## 6 DISCUSSION

Food‐related cues can drive eating in the absence of hunger by enhancing the motivation to consume food and overriding internal regulatory signals that control energy intake. The current studies identified a necessary role for the LHA feeding signal MCH in CPF, whereby hM4Di‐mediated inhibition of LHA MCH cells disrupted CPF. Furthermore, LHA MCH expressing cells can also augment CPF during acute optogenetic stimulation, as was demonstrated by increased licking for sucrose during CS+ stimulated compared to non‐stimulated trials in ChR2 mice. These latter optical manipulations also enhanced other appetitive behaviors, including entries to the food cup during CS+ presentations.

Multiple studies have characterized a role for MCH as an important orexigenic signal such that ICV administration promotes food intake and weight gain,^9^, ^30^ whereas antagonism of its receptor, MCH‐1R, reduces intake and provides resistance against dietary obesity.^31^, ^32^ MCH‐expressing cells are found throughout the rostral‐caudal gradient of the LHA^33^ and project widely throughout the brain including to the basolateral amygdala and medial prefrontal cortex^16^; regions that disrupt CPF when lesioned in rats.^3^, ^34^, ^35^ It should be noted; however, that these regions are thought to drive learned overeating behavior through top‐down control over the LHA.^36^ An additional mechanism could reflect LHA MCH cells that project to the NAc where they preferentially target the shell region^37^ and can modulate activity of medium spiny neurons through phosphorylation of AMPA glutamate receptors.^38^, ^39^ The NAc is critical for integrating motivational and reward‐related signals to drive appetitive behaviors,^40^ and direct infusion of MCH into the NAc promotes hedonic taste reactivity responses to a sweet‐tasting stimulus.^41^ Furthermore, acute optogenetic activation of LHA MCH cells projecting to the NAc leads to the development of food preferences associated with laser stimulation.^42^ It would be of interest to examine whether this subpopulation of MCH cells underlies the effects on CPF in the current study. More generally, our findings are consistent with the idea that LHA MCH cells promote reward‐based feeding behaviors^43^, ^44^ and that global deletion of MCH‐1R disrupts CPF.^21^ The current studies extend our understanding of LHA MCH as a critical feeding signal that both drives and is needed for cue‐evoked feeding behaviors in the absence of metabolic need.

Sights, smells or sounds associated with food can enhance food preferences and trigger overeating behaviors in the absence of hunger.^45^, ^46^, ^47^ To account for these effects in rodents, it has been suggested that CPF results from the acquisition of motivational value to food‐related stimuli, which drive food‐specific incentive motivation.^48^ Alternatively, using an analysis of licking microstructure to separately observe pre‐ and post‐ingestive variables controlling meal intake,^28^ we have shown that CPF can prolong the size of licking bursts,^49^ which could reflect an increase in hedonic evaluation or the perceived orosensory properties of the sucrose solution and thus result in increased food intake.^27^, ^28^, ^50^ In the current study, during CPF testing, CS+ presentation enhanced the number of licking bursts initiated, a measure that reflects the effects of post‐ingestive negative feedback as fluid accumulates in the gastrointestinal tract^51^ and/or the incentive value of the food stimulus.^49^ The increase in CS+ evoked burst number occurred even though chemogenetic inhibition of LHA MCH cells prevented CPF, suggesting that the initiation of individual bursts of licking occurred independently of MCH‐mediated mechanisms. Accordingly, the impairment in CPF observed here reflected a significant reduction in burst size in CNO‐treated mice, which, as mentioned, could reflect a CS+ evoked increase in the orosensory properties of the sucrose solution^52^ or alternatively pauses in licking resulting from a lack of contact of the tongue with the lickometer due to the expression of a lateral tongue protrusion.^53^, ^54^ This latter response has been associated with the ‘liking’ of a tastant and is thought to indicate an increase in the hedonic affective reactions to these stimuli.^55^ Collectively, our findings support the idea that CPF drives overeating by enhancing the orosensory and/or hedonic taste properties of food, an effect that requires intact LHA MCH cell signaling.

Although our findings clearly implicate LHA MCH cells in CPF there are several alternative considerations that should be addressed. Firstly, optogenetic stimulation of LHA MCH neurons appeared to have a broader impact on appetitive behaviors including enhancing entry rate to the food cup. This finding is consistent with the suggestion that MCH‐expressing cells promote the rewarding features of food‐related procurement and consummatory behaviors.^20^, ^42^, ^43^, ^44^ The extent of the influence of a more general increase in appetition over the reported CPF effects could further be disentangled using reinforcer‐specific CPF paradigms (e.g.,^56^). Nevertheless, certain features of our findings are more in line with a food‐specific CPF effect as chemogenetic disruption selectively disrupted licking measures related to the orosensory (but not motivational) properties of the sucrose and did not significantly impact food approach behaviors. Secondly, the DREADD study did not include administration of CNO in non‐hM4Di animals, which would have allowed for the confirmation that CNO administration alone did not impact CPF. CNO has been shown to reverse metabolize to clozapine, producing undesired effects, including over serotonergic, cholinergic and dopaminergic systems.^57^, ^58^, ^59^ Conceivably, the disruption in CPF following CNO administration could reflect these off‐target effects; however, it is important to note that these effects are typically revealed at CNO concentrations (e.g., >1 mg/kg) higher than that used in the current study. Thirdly, due to exclusion following confirmation of insufficient viral expression, the sample sizes for these studies were low, limiting the ability to confirm distributional assumptions of the data and the statistical power associated with the discussed inferential conclusions. Finally, our studies exclusively used male mice, yet significant sex differences in how MCH impacts feeding and other reward‐related behaviors have been noted^42^, ^60^; thus, future studies should examine whether female Tg(Pmch‐Cre) mice are similarly impacted by chemogenetic and optogenetic manipulations to LHA MCH cells.

LHA MCH cells have received significant interest due to their role in energy intake, metabolism, and as a potential tool to treat obesity.^9^, ^11^, ^30^ The current studies provide significant insight into the behavioral and neuronal mechanisms governing overeating behaviors. CPF appears to promote the orosensory taste features of food, which leads to an increase in feeding behavior outside of metabolic need. This capacity for food cues to augment intake in this manner requires intact LHA MCH neuronal signaling. Given that food‐related cues can drive feeding behaviors and food preferences in human adults and children^45^, ^46^, ^47^, ^61^ our findings emphasize the importance of continued study of the MCH system in governing these effects.

## AUTHOR CONTRIBUTIONS

**Lauren M. Raycraft:** Investigation; writing – review and editing. **Bingxin Mo:** Investigation; writing – review and editing. **Allison Doneth:** Investigation. **Jenna Lee‐Tanner:** Investigation; writing – review and editing. **Alexander W. Johnson:** Conceptualization; investigation; methodology; writing – review and editing; visualization; formal analysis.

## CONFLICT OF INTEREST STATEMENT

The authors declare no conflicts of interest.

## Data Availability

The data that support the findings of this study are available from the corresponding author upon reasonable request.
